# Fraxetin Targeting to Sortase A Decreases the Pathogenicity of *Streptococcus agalactiae* to Nile Tilapia

**DOI:** 10.3390/ani14091337

**Published:** 2024-04-29

**Authors:** Jing Dong, Yuze Zhang, Qiuhong Yang, Yongtao Liu, Shun Zhou, Xiaohui Ai

**Affiliations:** 1 Yangtze River Fisheries Research Institute, Chinese Academy of Fishery Sciences, Wuhan 430223, China; 2 College of Food Science and Engineering, Bohai University, Jinzhou 121010, China

**Keywords:** *Streptococcus agalactiae*, sortase A, fraxetin, tilapia, anti-virulence

## Abstract

**Simple Summary:**

Tilapia is the second-most-farmed fish worldwide which plays a critical role in providing high-quality proteins to human. However, infections caused by *S. agalactiae* result in huge economic losses. Moreover, the spreads of antibiotic resistance restrict the use of antibiotics in treating *S. agalactiae* infections. Here, we tried to develop anti-*S. agalactiae* drugs by inhibiting SrtA to overcome infections caused by resistant bacterial strains.

**Abstract:**

Sortase A (SrtA) is responsible for anchoring surface proteins to the cell wall, and has been identified as a promising target developing anti-infective drugs of Gram-positive bacteria. The aim of the study was to identify inhibitors of *Streptococcus agalactiae* (*S. agalactiae*) SrtA from natural compounds to overcome the spread of antibiotic resistance in aquaculture. Here, we found that the MIC of fraxetin against *S. agalactiae* was higher than 256 μg/mL, indicating that fraxetin had no anti- *S. agalactiae* activity. But fraxetin could dose-dependently decrease the activity of SrtA *in vitro* at concentrations ranging between 4–32 μg/mL by a fluorescence resonance energy transfer (FRET) assay. Moreover, the inhibition of SrtA by fraxetin decreased the anchoring of surface proteins with the LPXTG motif to the cell wall by detecting the immunofluorescence change of serine-rich repeat protein 1 (Srr1) on the bacterial cell surface. The results of fibronectin binding and cell adhesion assays indicated that fraxetin could significantly decrease the adhesion ability of *S. agalactiae* in a dose-dependent manner. The results were further proven by immunofluorescence staining. Animal challenge results showed that treatment with fraxetin could reduce the mortality of tilapia infected with *S. agalactiae* to 46.67%, indicating that fraxetin could provide a significant amount of protection to tilapia by inactivating SrtA. Taken together, these findings provided a novel inhibitor of *S. agalactiae* SrtA and a promising candidate for treating *S. agalactiae* infections in aquaculture.

## 1. Introduction

Tilapia, distributed in over 135 countries, has become the second-most-farmed fish all over the world [1]. The increasing demands of tilapia have led to the fast growing of the industry. According to a previous study, Asian countries, particular China and Southeast Asian countries, provided over 70% of tilapia products to the world’s consumers [1]. However, bacterial diseases have become the main concerns affecting the healthy development of the tilapia industry [2]. *Streptococcus agalactiae* (*S. agalactiae*) is a common bacterial pathogen which is responsible for a range of bacterial infections in aquatic fish, particular tilapia. The outbreak of *S. agalactiae* infections could result in economic losses of over 40 million dollars per year [3,4]. In aquaculture, antibiotics are the main measure dealing with bacterial diseases, including streptococcosis. Due to the occurrence of antibiotic resistance and residues in aquatic products, the applications of antibiotics in aquaculture were limited [5,6]. Thus, there is an urgent need to develop drugs with novel strategies for battling *S. agalactiae* infections in aquaculture.

With the increasing knowledge of bacterial infection processes, studies have shown that surface proteins mediated adhesion, and virulence factors play critical roles in the pathogenicity [7,8]. Therefore, strategies targeting bacterial adhesion and virulence factors have become the major approaches screening inhibitors against bacterial infections to alternate antibiotics. Sortase A (SrtA) is a transpeptidase enzyme which is widely distributed in Gram-positive bacteria [9]. SrtA can anchor proteins related to the virulence and adhesion to the cell surface with LPXTG motifs and it has been proven that SrtA is an ideal target for identifying anti-virulence drugs [10,11]. A previous study demonstrated that an SrtA knockout *S. agalactiae* strain could not anchor surface proteins to the cell wall and the adhesion ability of the strain was significantly reduced in several tested cell lines [12]. Moreover, serine-rich (Srr) proteins attached to the cell surface by SrtA played a critical role in the pathogenicity of *S. agalactiae* [13]. Taken together, all of the factors mentioned above satisfied the demand of SrtA as a target for anti-SrtA therapy [14]. Therefore, SrtA was selected as the target for screening inhibitors against *S. agalactiae*.

**Figure 1:**
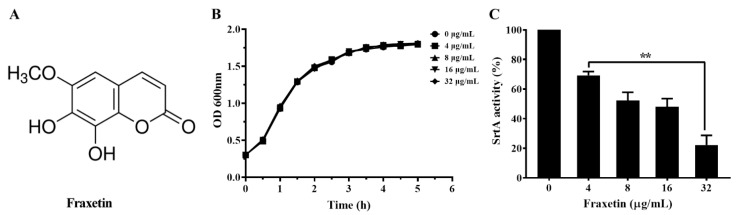
Fraxetin reduced the activity of SrtA. (**A**) Chemical structure of fraxetin. (**B**) Impact of fraxetin on bacterial growth determined by growth curves assay; values in growth curves were the averages of three independent assays. (**C**) Inhibition of SrtA activity by fraxetin. The results were mean ± SD of three independent assays; ** indicated *p* < 0.01.

Herbal medicines and their chemical components were widely used in treating infectious diseases of human, livestock, and poultry in China and some Asian countries [15]. Moreover, herbal medicines have been chosen as alternatives of antibiotics in treating diseases in aquaculture [16,17]. Fraxetin (Figure 1A), isolated from *Fraxinus rhynchophylla*, belonging to the coumarin derivative, has a number of biological activities, such as anti-oxidative, anti-inflammatory, anti-bacterial, and anti- tumor activities [18,19]. Here, we found that fraxetin could directly reduce the activity of SrtA and decrease the pathogenicity of *S. agalactiae* both *in vitro* and *in vivo*.

## 2. Materials and Methods

### 2.1. Bacterial Strain and Reagents

*S. agalactiae* strain PBSA0903, a sequence type (ST-7) strain isolated from tilapia, was provided by Prof. Weiliang Guo of Hainan University [20]. The bacterial strain was cultured in THB medium at 37 °C. Recombinant SrtA*_Δ82_*, a truncated SrtA lacking the N-terminal transmembrane domain [N_1–82_], was overexpressed and purified as our previous protocols [20]. The fluorescent substrate Dabcyl-QALPETGEE-Edans for determining the activity of SrtA*_Δ82_* was obtained from GL Biochem. Fraxetin (CAS No. 574-84-5) with a purity of 98% was a commercial product from Sichuan Vicky Biotechnology Co., Ltd. (Chengdu, China) and was dissolved in DMSO at a concentration of 40.96 mg/mL as a stock solution for *in vitro* studies. For animal study, fraxetin was dissolved in 0.5% DMSO.

### 2.2. Minimal Inhibitory Concentration (MIC) Determination

The MIC of fraxetin against *S. agalactiae* PBSA0903 was determined by the micro-broth dilution method in a 96-well microplate with the accordance of CLSI [21]. In brief, fraxetin was two-fold diluted by THB in a 96-well microplate at concentrations ranging from 512 to 1 μg/mL of 100 μL. Bacterial cells were acquired by centrifugation after being cultured at 37 °C to the mid-log phase. After washing with sterile PBS, McFarland standards were used to adjust the bacterial cells to a density of 1.5 × 10^5^ CFU/mL in THB medium. Then, 100 μL of bacterial cells were added to each well, and the plate was further incubated at 37 °C for 16–18 h. The MIC was defined as the lowest concentrations which completely inhibited the growth of the bacterium.

### 2.3. Growth Curves Assay

The effect of fraxetin on bacterial growth in 5 h was evaluated by a visible spectrophotometry. The overnight bacterial culture was sub-inoculated into a fresh THB medium at a ratio of 1: 100. Then, the suspension was further cultured at 37 °C until the optical density of 600 nm (OD_600nm_) reached 0.3. The inoculum was divided into 5 flasks with volumes of 20 mL, and fraxetin at final concentrations ranging from 4–32 μg/mL was added to each flask. DMSO was added into drug-free group to determine the effect of DMSO on bacterial growth. The growth of the cultures with or without fraxetin was determined by measuring the OD_600nm_ every 30 min.

### 2.4. Inhibition of SrtA Activity with Fraxetin

The inhibitory effect of fraxetin against SrtA*_Δ82_* was determined using fluorescence resonance energy transfer (FRET) method by a Synergy 2 microplate reader. Dabcyl-QALPETGEE-Edans was employed as fluorescently self-quenched peptides, tagged with Edans as the fluorophore, with Dabcyl as the quencher. The reaction was conducted in a 96-well black plate with a final volume of 300 μL. SrtA*_Δ82_* at 5 μM was firstly incubated with fraxetin at concentrations ranging from 4 to 32 μg/mL at 37 °C for 30 min. Then, the fluorescent substrate at 10 μM was added to each well and further incubated at 37 °C for 1 h. The fluorescence was determined after adding the substrate immediately and 1 h after incubation with an excitation at 350 nm and an emission at 520 nm. DMSO was added to drug-free control group and was defined as negative control.

### 2.5. Immunofluorescence Staining of Srr1 on Cell Surface

Immunofluorescence staining was performed according to a previous study with some modifications [13]. Briefly, *S. agalactiae* PBSA0903 was cultured in THB medium to OD_600nm_ of 0.3 with or without fraxetin. Bacterial cells were re-suspended to OD_600nm_ of 1.0 after being washed 3 times with sterile PBS; then, 50 μL of the bacterial cells were applied to glass coverslips and incubated for 15 min at 25 °C. The glass coverslips were washed with PBS and fixed with 0.5% paraformaldehyde. After being blocked with 5% BSA, cells were incubated with an anti-Srr1 polyclonal antibody (prepared in our laboratory) for 2 h, and then incubated with an Alexa Flour^®^ 488 goat anti-rabbit IgG (Yeasen, Shanghai, China) for 30 min. After washing, the slides were mounted in mountant plus DAPI to stain bacterial DNA. Bacterial cells were captured with a fluorescent microscope.

### 2.6. Fibronectin Binding Assay

The impact of fraxetin on *S. agalactiae* binding to human fibronectin was evaluated according to a previously reported study [22]. Briefly, a 96-well cell plate was coated with 100 μL fibronectin at a final concentration of 2 μg/mL at 4 °C overnight. The plate was blocked with 3% BSA at 37 °C for 1 h after being washed with PBS for three times. To prepare bacterial suspension, *S. agalactiae* PBSA0903 was cultured in THB medium with fraxetin at concentrations of 4, 8, 16, and 32 μg/mL. DMSO was added in drug-free group as control. Bacterial cells were harvested by centrifugation until OD_600nm_ reached 0.5; then, cell density was adjusted to OD_600nm_ of 1.0 after being washed with sterile PBS. 100 μL of bacterial suspension described above was loaded to each well pre-coated with fibronectin and further incubated at 37 °C for 2 h. The plate was washed to remove the unattached bacterial cells and formaldehyde was added to fix cells attached with fibronectin. After being washed with PBS, bacterial cells in each well were stained with 0.5% crystal violet at 37 °C for 10 min. The binding ability of *S. agalactiae* after being treated with fraxetin was determined by measuring the values of OD_570nm_ of each well after the addition of 30% acetic acid.

### 2.7. Bacterial Adhesion Assay

A549 cells were used to determine the adhesion ability of *S. agalactiae* PBSA0903 after a co-incubation with fraxetin. The assay was carried out as described previously [12]. Briefly, A549 cells were cultured in DMEM plus 10% FBS at 37 °C with 5% carbon dioxide. Cells were digested with trypsin and the density was adjusted to about 4 × 10^5^ cells/mL using a hemocytometer. Cell cultures at a volume of 1 mL were added to each well of a 24-well cell plate and incubated for 16–18 h. An overnight *S. agalactiae* PBSA0903 culture was sub-inoculated into 100 mL fresh THB medium and immediately divided into 5 flasks at a volume of 20 mL. Then, fraxetin at final concentrations of 4, 8, 16, and 32 μg/mL was added to each flask. DMSO was served as drug-free group. The suspensions were further incubated at 37 °C to OD_600nm_ of 0.5. Bacterial cells were acquired by centrifugation and were washed with sterile PBS 3 times. Bacterial cells were re-suspended in DMEM and were added into A549 cells with a multiplicity of infection of 20. After centrifugation, cells were further incubated for 2 h post infection. Unattached bacterial cells were removed, washing the plate 3 times with PBS. Then, ice-cold water was added to each well and pipetted repeatedly to release the bacterium from A549 cells. The numbers of adherent bacterial cells were determined by plating cell lysis to THB agar plates after appropriate dilutions.

Moreover, the adherence of *S. agalactiae* to A549 cells were evaluated by immunofluorescence staining according to previous protocols [23]. A549 cells were seeded onto glass coverslips in a 24-well plate in DMEM and were cultured overnight at 37 °C with 5% carbon dioxide; infection protocols were the same as described above. A549 cells after a co-incubation with 32 μg/mL fraxetin-treated *S. agalactiae* PBSA0903 were firstly washed with pre-warmed PBS three times and fixed with 4% paraformaldehyde dissolved in PBS. Cells were blocked with 5% BSA for 30 min after permeabilized with 0.3% Triton X-100 in PBS for 3 min. Then, cells were co-incubated with a FITC-conjugated anti-GBS polyclonal antibody (Thermo Fisher Scientific, Waltham, MA, USA) and Alexa Fluor plus 647 phalloidin (Yeasen, Shanghai, China) for 1 h to label *S. agalactiae* and F-actin, respectively. Nuclei was stained with mountant containing DAPI after being washed with PBS plus 0.1% Triton X-100. Images were captured by a Nikon C2^+^ confocal microscope system.

### 2.8. Challenge Test

Nile tilapia was used to determine the therapeutic effect of fraxetin against *S. agalactiae* infection. The protocols of animal studies were approved by the Animal Welfare and Research Ethics Committee at Yangtze River Fisheries Research Institute. 90 healthy Nile tilapia (100 ± 10 g) were divided into 3 groups; each group contained 3 biological repeats. Fish were maintained in glass tanks of 100 L with dissolved oxygen of 5.5–7 mg/L at 30 ± 2 °C for 7 days before use. *S. agalactiae* PBSA0903 was cultured in THB medium to OD_600nm_ of 1.0; then, bacterial cells were harvested and re-suspended in PBS at a density of 1.5×10^8^ CFU/mL. Fish in positive control and fraxetin-treated groups were infected with *S. agalactiae* by an intraperitoneal injection of bacterial suspension at volumes of 200 μL after being anesthetized by 20 mg/L tricaine methanesulfonate (MS-222) to establish the infection model, while fish in negative control group were injected with sterile PBS. Fish in fraxetin-treated group were orally given 50 mg/kg fraxetin by a gavage needle, while fish in positive and negative controls were given the same volume of 0.5% DMSO. The course was maintained for 3 days with 12 h intervals. Deaths in each tank were recorded for 10 d to determine the protective effect of fraxetin against *S. agalactiae* infection.

### 2.9. Statistical Analysis

SPSS 13.0 software was used to determine the statistical significance of *in vitro* data by t-test method, while the mortalities were analyzed by Kaplan–Meier estimates and log-rank tests by GraphPad software V8.

## 3. Results

### 3.1. Impact of Fraxetin on Bacterial Growth

The MIC of fraxetin against *S. agalactiae* PBSA0903 was higher than 256 μg/mL; the result indicated that fraxetin had no anti-bacterial activity against *S. agalactiae*. Moreover, the growth curves of *S. agalactiae* PBSA0903 co-cultured with fraxetin were determined. As shown in Figure 1B, the growth curves of *S. agalactiae* PBSA0903 with fraxetin at concentrations ranging from 4 to 32 μg/mL were similar to that of *S. agalactiae* PBSA0903 with DMSO, indicating that fraxetin had no influence on bacterial growth. Altogether, the findings demonstrated that fraxetin had no role on bacterial growth under our experimental conditions as described above.

### 3.2. Fraxetin Decreased the Peptidase Activity of SrtA

According to the results of the FRET assay, we found that fraxetin could dose-dependently inhibit the peptidase activity of SrtA. As shown in Figure 1C, the relative activity of SrtA was decreased to 69.29 ± 3.14, 52.18 ± 3.64, 47.99 ± 6.75, and 22.20 ± 5.06% when co-incubated with fraxetin at concentrations of 4, 8, 16, and 32 μg/mL, respectively. According to the results, fraxetin could significantly reduce the catalytic activity of SrtA at concentrations ranging from 4 to 32 μg/mL. The 50% inhibitory concentration of fraxetin against SrtA activity was about 77.53 μM.

### 3.3. Fraxetin Influenced the Anchoring of Srr1 to Cell Surface

**Figure 2:**
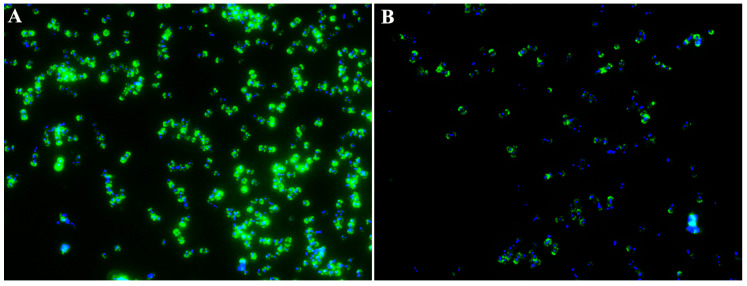
Changes of Srr1 exhibited at bacterial cell surface after treatment with fraxetin. (**A**) Bacterial cells without any treatment. (**B**) Bacterial cells after a co-incubation with fraxetin at 32 μg/mL. Srr1 was stained green by anti-Srr-1 antibody and Alexa Flour^®^ 488 goat anti-rabbit IgG; bacterial DNA was stained blue with DAPI.

Srr1, a virulence factor contributing to the full virulence of *S. agalactiae*, is one of the cell-wall-anchoring proteins containing the LPXTG motif at the carboxyl terminus. A previous study demonstrated that an *S. agalactiae* strain lacking the *srtA* gene could not anchor Srr1 to the cell surface [13]. Thus, we determined the impact of fraxetin on the anchoring of Srr1 on the cell surface by immunofluorescence staining. As shown in Figure 2A, *S. agalactiae* PBSA0903 without fraxetin treatment showed a strong signal after being probed with the anti-Srr1 antibody. However, *S. agalactiae* after being co-cultured with 32 μg/mL fraxetin showed an obvious decrease in green fluorescence (Figure 2B), indicating the reduction of Srr1 on cell surface. The result demonstrated that fraxetin could affect the location of Srr1 on the bacterial surface by interference with SrtA activity.

### 3.4. Fraxetin Affected the Binding Ability of S. agalactiae to Fibronectin

**Figure 3:**
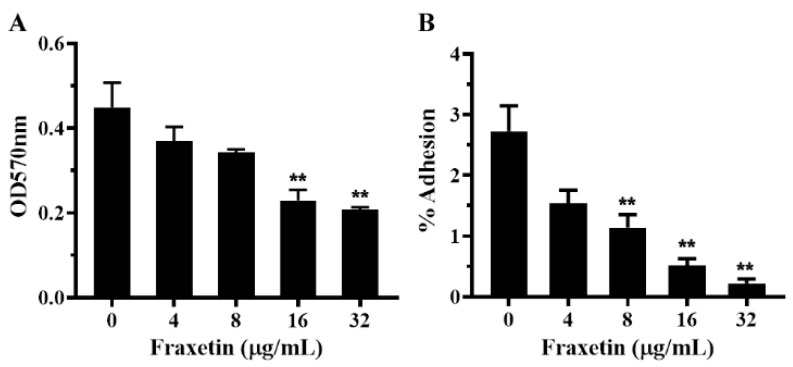
The inhibitory effect of fraxetin on bacterial adhesion. (**A**) Fraxetin decreased the binding of *S. agalactiae* to fibronectin. (**B**) Fraxetin affected the adhesion of *S. agalactiae* to A549 cells. The results were mean ± SD of three independent assays; ** indicated *p* < 0.01.

It is known that adherence to host tissues is an essential step to establish infections and cause diseases for bacterial pathogens [24]. Surface proteins anchored by SrtA play key roles in bacterial adherence and infections for most Gram-positive bacteria [25]. Moreover, Lalioui et al. demonstrated that the *S. agalactiae* strain lacking an *srtA* gene resulted in a significant reduction in the binding to human fibronectin [12]. Thus, the inhibitory effect of fraxetin against the adherence of *S. agalactiae* was determined by the fibronectin binding assay. As shown in Figure 3A, fraxetin could inhibit the binding of *S. agalactiae* to fibronectin in a dose-dependent manner. The relative binding rate decreased to 82.60 ± 3.75, 77.22 ± 9.70, 51.73 ± 10.26, and 46.48 ± 5.48%, plus fraxetin of 4, 8, 16, and 32 μg/mL, while the fraxetin-free group served as 100% binding. Statistical significance was observed when the concentration of fraxetin reached 16 μg/mL. The finding indicated that fraxetin could inhibit the binding ability of *S. agalactiae* to fibronectin by inhibiting SrtA activity.

### 3.5. Fraxetin Inhibited the Adhesion of S. agalactiae to A549 Cells

**Figure 4:**
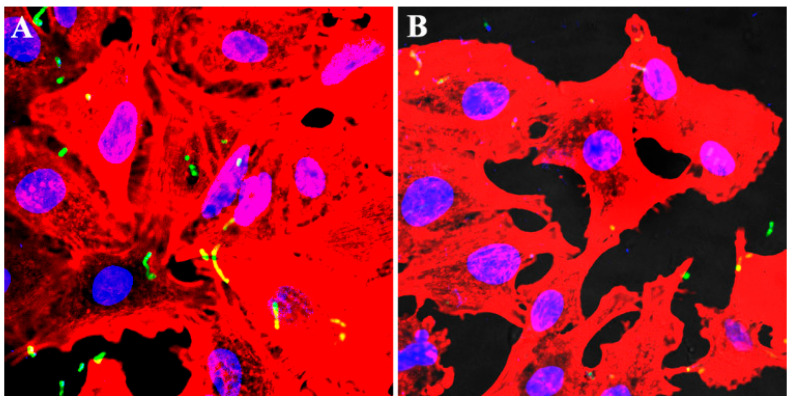
Labeling of *S. agalactiae* PBSA0903 without (**A**) or with fraxetin (**B**) treatment in A549 cells after infection. *S. agalactiae* PBSA0903 were stained green, while actin and nuclei were red and blue, respectively.

An adhesion assay was carried out to determine the inhibitory effect of fraxetin against the bacterial adhesion to A549 cells. According to the results, *S. agalactiae* with the presence of fraxetin could reduce the adhesion to A549 cells in a dose-dependent manner (Figure 3B). The adhesion rate of the fraxetin-free group was about 2.73 ± 0.42%, while the adhesion rate was 1.54 ± 0.22, 1.14 ± 0.21, 0.51 ± 0.11, and 0.22 ± 0.08% after being treated with fraxetin at the indicated concentrations, respectively. The adhesion was remarkably inhibited by the addition of fraxetin at concentrations from 8 to 32 μg/mL (Figure 3B). Moreover, the adhesion ability of *S. agalactiae* with or without fraxetin treatment was then confirmed by immunofluorescence staining. As shown in Figure 4, *S. agalactiae* PBSA0903 adhered to the A549 cell surface showed a green color after staining by an anti-GBS FITC-conjugated antibody, while the nuclei and F-actin showed a blue and red color, respectively, after labeling by DAPI and Alexa Fluor plus 647 phalloidin. *S. agalactiae* after treatment with 32 μg/mL fraxetin showed a visible decrease in cell surface (Figure 4B). Taken together, bacterial cells after a co-incubation with fraxetin could reduce the adhesion of *S. agalactiae* to A549 cells, demonstrating that blocking the function of SrtA could affect the adhesion of *S. agalactiae*.

### 3.6. Fraxetin Protected Tilapia against S. agalactiae Infection

**Figure 5:**
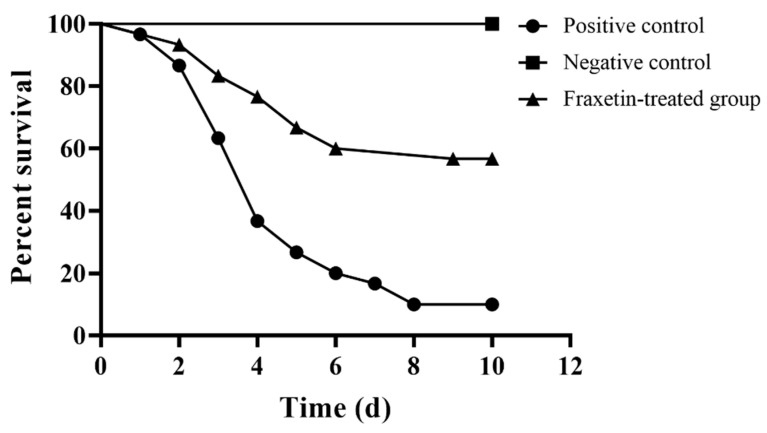
Fraxetin alleviated the mortality of tilapia infected with *S. agalactiae*. Healthy Nile tilapia were intraperitoneally injected with *S. agalactiae* to establish the infection model; then, fish in fraxetin-treated group were treated with 50 mg/kg fraxetin, while fish in positive and negative control groups were treated with 0.5% DMSO. Fish were monitored for 10 days and statistical significance was determined by log-rank test (*p* < 0.0001). Mean values of three independent assays are shown in the figure.

The results achieved above meant that fraxetin might have a potential therapeutic effect against *S. agalactiae* infection in tilapia. Therefore, an infection model was designed to determine the protective effect of fraxetin to tilapia challenged with *S. agalactiae*. Deaths were observed in 24 h post infection in the positive control group, while they were observed on the second day for the fraxetin-treated group (Figure 5). The results indicated that fraxetin could delay the occurrence of death. As shown in Figure 5, the survival rate of fish in the positive control group was 10%, while 56.67% of fish in the fraxetin-treated group survived. Treatment with 50 mg/kg fraxetin could significantly reduce the mortality of fish post infection with *S. agalactiae* after being analyzed by the log-rank test. All fish in the negative control group were alive during the experimental period. The findings demonstrated that fraxetin could provide protection to tilapia against *S. agalactiae* infection by the interference of the activity of SrtA.

## 4. Discussion

Antibiotics, one of the greatest inventions in the 20th century, have been used for more than 70 years in treating bacterial infections and have saved millions of lives [26]. Moreover, antibiotics were widely used in livestock farming and aquaculture as growth promoting and anti-bacterial agents. The introduction of antibiotics to aquaculture decreased economic losses caused by bacterial diseases and promoted the healthy development of the industry. However, the misuse of antibiotics in aquaculture resulted in antibiotic resistance and residues in aquatic products, which might bring potent risks to human [27]. We have entered the post-antibiotic era, meaning that bacterial infections are difficult to treat with antibiotics. Consequently, there is a pressing need to investigate novel strategies or therapeutics for dealing with antibiotic resistance. Although the susceptibility of penicillin was reduced in some clinical strains, penicillin is still considered as the main drug dealing with *S. agalactiae* infections in human [28,29,30]. However, studies have reported that *S. agalactiae* strains isolated from aquaculture have a severe drug resistance to antibiotics which extremely limits the treatment of *S. agalactiae*-associated diseases [31,32,33]. Thus, SrtA was introduced as a promising target to screen drugs based on an anti-virulence strategy in the present study.

Previous studies demonstrated that fraxetin had anti-bacterial activities against several bacteria. Liu et al. found that fraxetin could inhibit the growth of *Escherichia coli* by disturbing protein synthesis with an MIC of 40 μg/mL [34]. Wang et al. systematically determined the inhibitory effect of fraxetin against *Staphylococcus aureus* (*S. aureus*); the results showed that fraxetin could inhibit the proliferation of *S. aureus* by destroying nucleic acid and protein synthesis [19]. Zhu et al. demonstrated that fraxetin had anti-*Aeromonas hydrophila* (*A. hydrophila*) activity with an MIC of 128 μg/mL [35]. Liu et al. found that the MICs of fraxetin against group A *Streptococcus* and group B *Streptococcus* were 128 and 320 μg/mL, respectively [36]. Here, we found that the MIC of fraxetin against *S. agalactiae* was higher than 256 μg/mL, which was similar to the results of Liu et al. [36]. Although fraxetin could not be used as an anti-*S. agalactiae* agent, we found that fraxetin could inactivate the catalytic activity of SrtA *in vitro*. Several natural compounds have been identified as SrtA inhibitors of *Streptococcus*, such as curcumin, alnustone, astilbin, and quercetin [37,38,39,40]. However, there was little information on inhibitors against the SrtA of *S. agalactiae*. Lila et al. found that *S. agalactiae* stain lack of the a *srtA* gene could decrease the adhesion of *S. agalactiae* to epithelial cells, but had no impact on the pathogenicity of the strain determined by a mice infection model [12]. Mistou showed that *S. agalactiae*’s lack of the *srtA* gene lost the ability of anchoring Srr1 to the bacterial cell surface by the dot-blot assay; the finding revealed that SrtA might influence the pathogenicity of *S. agalactiae* by anchoring Srr1 or some other proteins with the LPXTG motif [13]. Here, we found that the immunofluorescence of Srr1 in the cell surface showed a visible decrease after fraxetin treatment, which was similar to that reported by Mistou et al. [13].

Fraxetin was often known as an agent with anti-oxidative and anti-inflammatory activities; only several studies reported that fraxetin could be developed as an anti-virulence drug against bacterial infections [41]. Shi et al. found that fraxetin could inhibit the transcription of the *hilD*, *hilC*, and *rstA* genes of the *Salmonella enterica* serovar Typhimurium T3SS and resulted in a decrease in pathogenicity in a mice model [42]. Zhu et al. studied the inhibitory effect of fraxetin against the virulence of *A. hydrophila in vitro*; the results showed that fraxetin could decrease the activity of lipase and protease in bacterial supernatants at concentrations much lower than the MIC [35]. Moreover, Zhu et al. found that fraxetin could increase the level of non-specific immunity and anti-oxidative ability, which might help to reduce the mortality of *Megalobrama amblycephala* after being challenged with *A. hydrophila* [43]. Thus, it is reasonable to believe that fraxetin might have a similar effect to tilapia after being challenged with *S. agalactiae*. Taken together, fraxetin is a promising candidate for preparing an anti-*S. agalactiae* drug for tilapia farming in future.

## 5. Conclusions

In conclusion, fraxetin without anti-*S. agalactiae* activity was identified as a *S. agalactiae* SrtA inhibitor. Moreover, fraxetin could reduce the adhesion and pathogenicity of *S. agalactiae* both *in vitro* and *in vivo*. To our knowledge, fraxetin as an inhibitor of SrtA was reported for the first time. The findings here offered a new candidate for treating *S. agalactiae* infections in aquaculture which might be developed as a fishery drug in future. In addition, the study proved that it is feasible to identify drugs against *S. agalactiae* in aquaculture using SrtA as the target.

## Data Availability

The data that support the findings of this study are available from the corresponding author upon reasonable request.
